# Linking Lysosomal Enzyme Targeting Genes and Energy Metabolism with Altered Gray Matter Volume in Children with Persistent Stuttering

**DOI:** 10.1162/nol_a_00017

**Published:** 2020-08-01

**Authors:** Ho Ming Chow, Emily O. Garnett, Hua Li, Andrew Etchell, Jorge Sepulcre, Dennis Drayna, Diane Chugani, Soo-Eun Chang

**Affiliations:** 1 Department of Communication Sciences and Disorders, University of Delaware, Newark, DE; 2 Katzin Diagnostic & Research PET/MRI Center, Nemours/Alfred I. duPont Hospital for Children, Wilmington, DE; 3 Department of Psychiatry, University of Michigan, Ann Arbor, MI; 4 Department of Psychiatry, University of Michigan, Ann Arbor, MI; 5 Katzin Diagnostic & Research PET/MRI Center, Nemours/Alfred I. duPont Hospital for Children, Wilmington, DE; 6 Department of Psychiatry, University of Michigan, Ann Arbor, MI; 7 Gordon Center for Medical Imaging, Department of Radiology, Massachusetts General Hospital, Harvard Medical School, Boston, MA; 8 National Institute on Deafness and Other Communication Disorders, NIH, Bethesda, MD; 9 Department of Communication Sciences and Disorders, University of Delaware, Newark, DE; 10 Department of Psychiatry, University of Michigan, Ann Arbor, MI; 11 Cognitive Imaging Research Center, Department of Radiology, Michigan State University, East Lansing, MI; 12 Department of Communicative Sciences and Disorders, Michigan State University, East Lansing, MI

**Keywords:** longitudinal, lysosome, metabolism, stuttering, voxel-based morphometry

## Abstract

Developmental stuttering is a childhood onset neurodevelopmental disorder with an unclear etiology. Subtle changes in brain structure and function are present in both children and adults who stutter. It is a highly heritable disorder, and 12–20% of stuttering cases may carry a mutation in one of four genes involved in intracellular trafficking. To better understand the relationship between genetics and neuroanatomical changes, we used gene expression data from the Allen Institute for Brain Science and voxel-based morphometry to investigate the spatial correspondence between gene expression patterns and differences in gray matter volume between children with persistent stuttering (*n* = 26, and 87 scans) and their fluent peers (*n* = 44, and 139 scans). We found that the expression patterns of two stuttering-related genes (*GNPTG* and *NAGPA*) from the Allen Institute data exhibited a strong positive spatial correlation with the magnitude of between-group gray matter volume differences. Additional gene set enrichment analyses revealed that genes whose expression was highly correlated with the gray matter volume differences were enriched for glycolysis and oxidative metabolism in mitochondria. Because our current study did not examine the participants’ genomes, these results cannot establish the direct association between genetic mutations and gray matter volume differences in stuttering. However, our results support further study of the involvement of lysosomal enzyme targeting genes, as well as energy metabolism in stuttering. Future studies assessing variations of these genes in the participants’ genomes may lead to increased understanding of the biological mechanisms of the observed spatial relationship between gene expression and gray matter volume.

## INTRODUCTION

Fluid, effortless speech production forms the basis for communication and is considered a fundamental human ability. Stuttering significantly disrupts fluent speech production, often leading to negative psychosocial and economic consequences throughout life (Blumgart, Tran, & Craig, 2010; Craig, Blumgart, & Tran, 2009; Yaruss, 2010). Developmental stuttering typically has an onset in early childhood, affecting more than 5% of preschool-age children and persisting in about 1% of adults (Craig, Hancock, Tran, Craig, & Peters, 2002; Månsson, 2000; Yairi, & Ambrose, 2013). Persistent stuttering is highly heritable, with estimations of genetic contribution exceeding 80% in some studies (Dworzynski, Remington, Rijsdijk, Howell, & Plomin, 2007; Fagnani, Fibiger, Skytthe, & Hjelmborg, 2011; Ooki, 2005; Rautakoski, Hannus, Simberg, Sandnabba, & Santtila, 2012; van Beijsterveldt, Felsenfeld, & Boomsma, 2010). Genes causative of persistent stuttering have begun to be identified (Kang et al., 2010; Raza et al., 2015). To date, four such genes, designated *GNPTG*, *GNPTAB*, *NAGPA*, and *AP4E1* have been found, and they may cumulatively account for 12–20% of unrelated individuals with persistent stuttering (see Frigerio-Domingues & Drayna, 2017 for a comprehensive review).

This group of genes is known to play a role in lysosomal enzyme trafficking. *GNPTG*, *GNPTAB*, and *NAGPA* are involved in marking lysosomal hydrolases and several nonlysosomal proteins with a mannose-6-phosphate (M6P) tag that is important for intracellular trafficking (Barnes et al., 2011). Homozygous mutations in *GNPTG* and *GNPTAB* genes are known to cause the rare inherited lysosomal storage disorders Mucolipidosis types II and III (Kornfeld, 2001), which affect many parts of the body including the brain. However, in most of the cases, people who stutter only carry heterozygous mutations in these genes, and do not have the signs or symptoms typically seen in Mucolipidosis types II and III. *AP4E1* is a member of a family of adaptor proteins that are involved in vesicle formation and sorting member proteins for transporting lysosomal enzymes from the trans Golgi network to late endosomes and lysosomes (Dell’Angelica, Mullins, & Bonifacino, 1999). Mutations in *AP4E1* have been associated with hereditary spastic paraplegia and cerebral palsy (Abou Jamra et al., 2011; Kong et al., 2013). Why mutations in these genes can specifically affect the ability to produce fluent speech but not other cognitive or neurologic functions remains unknown. However, neuroimaging studies have shown that persistent stuttering is associated with subtle functional and anatomical anomalies (Chow & Chang, 2017; Etchell, Civier, Ballard, & Sowman, 2018; Garnett et al., 2018; Neef, Anwander, & Friederici, 2015).

### The Current Study

How genetic factors relate to brain anomalies in stuttering is not yet clear. To pursue this question, we compared the differences in spatial patterns of gray matter volume (GMV) in children with persistent stuttering with the regional expression of the four genes thus far associated with stuttering using data provided by the Allen Institute for Brain Science (AIBS; http://www.brain-map.org/). This approach has been used to reveal gene–brain relationships in a number of recent studies. A seminal study was published in 2015, in which the authors used gene expression data and resting-state fMRI to identify 136 genes associated with intrinsic functional networks in the brain (Richiardi et al., 2015). In another study, Ortiz-Terán et al. (2017) used a similar method to demonstrate that the neural reorganization in blind children measured by resting-state functional connectivity is associated with a set of known neuroplasticity-related genes. Moreover, this approach was also employed to study neurological disorders. For example, McColgan et al. (2017) identified genes associated with Huntington’s disease by comparing regional white matter loss and gene expression in patients with the disorder.

A core presumption of this current study is that the expression patterns of the genes known to be associated with stuttering, to a certain extent, reflect the pattern of anatomical anomalies in the disorder. While the genetic causes of stuttering are likely to be heterogeneous, their effects at the neuroanatomical level may be similar because the disorder affects speech production specifically. Moreover, in support of the previous argument, neuroimaging studies of people who stutter with unknown genetic status have shown some consistent results. In particular, different groups of researchers have independently demonstrated that the anisotropic diffusivity in the corpus callosum and the superior longitudinal fasciculus is decreased in people who stutter compared with matched controls (Neef et al., 2015). A similar presumption was made in a previous study showing that the expression of risk genes for schizophrenia were positively correlated with the anatomical disconnectivity defined by diffusion tensor imaging tractography in patients with schizophrenia, but not in patients with bipolar disorder (Romme, de Reus, Ophoff, Kahn, & van den Heuvel, 2017).

In our recent study, using a similar approach, we have shown that the pattern of functional connectivity differences between people who stutter and controls and the expression pattern of *GNPTG* are spatially correlated (Benito-Aragón et al., 2019). In the current study, we further examined whether the expression pattern of the genes associated with stuttering are correlated with the structural differences as reflected in the pattern of regional GMV. We hypothesized that the expression of the four stuttering-related genes from the AIBS data would be spatially correlated with the GMV differences in children with persistent stuttering. Moreover, we used gene set enrichment analysis to identify genes whose expression is highly associated with the GMV differences in order to explore the potential biological processes and pathways involved in stuttering. It is important to note that in these previous studies and our current study, because the genomes of the participants were not studied, the spatial relationship between brain measures and gene expression, if any, cannot provide evidence of a direct relationship between gene expressions and brain anomalies.

## MATERIALS AND METHODS

Participants were monolingual English speakers primarily recruited from the East Lansing, Michigan, area and within a surrounding 50-mile radius, as part of an ongoing longitudinal study conducted at Michigan State University. Subjects were between three and ten years old when they entered the study, and participated in 1 to 4 longitudinal visits, with an intervisit interval of approximately 12 months. All research procedures were approved by the Michigan State University Institutional Review Board, which follows the ethical standards described in the Belmont Report and complies with the requirements of the Federalwide Assurance for the Protection of Human Subjects from the United States Department of Health and Human Services. Written consent was obtained from all parents of the participating children, and assents were obtained from all children in verbal or written format depending on reading level. Children were paid a nominal remuneration, and were given small prizes (e.g., stickers) for their participation.

Participants were recruited via printed advertisements in newspapers and magazines, parent email listservs, study flyers, social media or online ads (e.g., Facebook, Craigslist), speech-language pathologist (SLP) referral, and word of mouth. A total of 226 children were contacted and screened for eligibility by an SLP on the study team. Of those screened, 128 were eligible and willing to participate in the study. Exclusion criteria for all children included (a) hearing loss, (b) a history of diagnosis of a developmental, psychological, neurological, or speech disorder, except for stuttering (e.g., autism spectrum disorder), (c) currently taking medication affecting central nervous functioning, (d) language impairment based on standardized tests (<−2 *SD*), (e) cognitive impairment based on standardized tests (<−2 *SD*), (f) bilingualism from a young age, (g) pregnancy, and (h) any type of metal implant or braces. Inclusion criteria for fluent controls included (a) no history of speech disorder at any time, and (b) stuttering-like disfluencies (SLDs) less than 3%. For children who stutter, they must have been stuttering for at least six months. The details of the standardized tests and behavioral evaluations are listed below.

### Standardized Tests and Speech Samples

All children were tested by trained, licensed speech language pathologists for cognitive, speech-language, and articulation abilities through a battery of standardized assessments, including the Wechsler Primary and Preschool Scale of Intelligence (3rd Ed.), for children 2:6–7:3 (Wechsler, 2002), the Wechsler Abbreviated Scale of Intelligence for children 7 and up (Wechsler, 1999), the Peabody Picture Vocabulary Test (PPVT-4) for receptive vocabulary ability (Dunn & Dunn, 2007), the Expressive Vocabulary Test (EVT-2; Williams, 2007), and the Goldman-Fristoe Test of Articulation 2 (GFTA-2; Goldman & Fristoe, 2000). The results of these tests are listed in Table 1. Further, all children performed within normal range on the Oral Speech Mechanism Screening Examination Revised (St Louis & Ruscello, 1987), which tests oral facial structure and function, as well as diadochokinetic rates. Participants’ phonological processing skills were not formally tested in this cohort, although clinicians made note of any phonological delay or processes based on their interaction with the child during conversation and test administration. All children were required to pass a hearing screening that was administered using an audiometer (Beltone model 119) at pure tone frequencies 500, 1,000, 2,000, and 4,000 Hz at the 20 dB threshold. The analyses of the above-mentioned tests were conducted according to instrument guidelines by trained, licensed SLPs.

**Table 1.** Demographics, intelligent quotient (IQ), and language test scores averaged across longitudinal visits for each participant

	Controls *n* = 44 (21 boys)		Persistent *n* = 26 (18 boys)		Recovered *n* = 17 (9 boys)	
	Mean (*SD*)	Range	Mean (*SD*)	Range	Mean (*SD*)	Range
Age at the first scan (years)	6.5 (2.0)	3.3–10.8	6.5 (1.9)	3.6–10.3	5.4 (1.9)	3.1–9.4
IQ^a^	114 (14.1)	84–144	106 (15.5)	81–138	106 (13.1)	88–128
PPVT-4^b^	119 (12.7)	95–141	110 (13.5)	86–146	114 (10.3)	93–131
EVT-2^c^	115 (11.8)	93–142	106 (12.2)	86–138	109 (9.2)	89–129
GFTA-2^d^	104 (6.6)	84–115	102 (4.2)	92–110	106 (7.3)	91–115
SSI-4^e^	-	-	21 (8.3)	12–48	13 (2.9)	7–19

^a^ Wechsler Primary and Preschool Scale of Intelligence (3rd Ed.) or Wechsler Abbreviated Scale of Intelligence). No significant difference between any two groups (*t* tests, *p* > 0.05).

^b^ The Peabody Picture Vocabulary Test (4th Ed.). Scores significantly higher in control than persistent groups (two-sample *t* tests, *p* < 0.05).

^c^ The Expressive Vocabulary Test (2nd Ed.). Scores significantly higher in control than both persistent and recovered groups (two-sample *t* tests, *p* < 0.05).

^d^ The Goldman-Fristoe Test of Articulation 2. Scores significantly higher in control than persistent groups (two-sample *t* tests, *p* < 0.05).

^e^ Stuttering Severity Instrument. Scores significantly higher in persistent and recovered groups (two-sample *t* tests, *p* < 0.05).

For study inclusion, participants had to score above −2 *SD* of the nominative mean on all standardized tests. The reason for using this threshold for inclusion was to ensure that we recruited a representative sample of children who stutter (CWS), who have been reported to exhibit greater comorbidity of articulation and language deficits and dissociated development among language areas (Choo, Burnham, Hicks, & Chang, 2016; Coulter, Anderson, & Conture, 2009). Nevertheless, in our sample, none of the subjects fell at or below −2 *SD* on any of the assessments, and only two from each group tested at or below −1 *SD* on the tests (1 CWS for GFTA and 1 CWS for PPVT; 2 controls on GFTA).

### Determination of Stuttering Status and Eventual Persistence versus Recovery

The Stuttering Severity Instrument (SSI-4; Riley, 2009), as well as offline analysis of recorded speech samples comprising both conversation (parent, clinician samples separately) and narrative samples elicited through storytelling using the wordless book, *Frog, where are you?* (Mayer, 1969), were used to conduct disfluency analysis, quantify the frequency of SLDs, note any physical concomitants of stuttering (e.g., tense lips, facial muscles during stuttering), and duration of stuttering blocks and prolongations. SSI-4 was used to examine frequency and duration of disfluencies occurring in the speech sample, as well as any physical concomitants associated with stuttering. These were incorporated into a composite stuttering severity rating (Riley, 2009). Children were determined to be in the stuttering group if they exhibited at least 3% SLDs, for example, part-word repetitions, prolongations, and blocks (Yairi, Ambrose, Paden, & Watkins, 2005), and scored at least in the “very mild” category based on the composite SSI-4 score. To confirm reliability of the SSI-4 scores, a random subset of the speech samples (25%) was rated by a second independent SLP. The intraclass correlation coefficient calculated based on the two SLPs’ ratings was 0.96, indicating high reliability. In borderline cases, expressed parental concern and clinician report were considered to confirm the diagnosis of stuttering. Children in the control group exhibited less than 3% SLDs, reported no family history of stuttering, and neither clinician nor parent reported any concern regarding stuttering.

CWS were further categorized as *recovered* (rCWS) or *persistent* (pCWS) based on their SSI-4 scores from two or more visits. A child was considered recovered if the SSI-4 score was 10 or below at the second visit or thereafter. A child was categorized as persistent if the composite SSI-4 score was higher than 10 at the second visit or thereafter. Both clinician and parent reports were required to be consistent with stuttering severity assessments in determining whether a child had recovered or was persistent. Three CWS were excluded because they were assessed only once and, therefore their final diagnoses could not be determined.

### Exclusion due to MRI Tolerance, Excess Head Movement and Incidental Findings

Thirty-three of 128 subjects did not successfully complete the MRI session for variable reasons (e.g., uneasiness in the MRI setting and anxiety detected during mock scanner training), leaving 50 CWS (20 girls and 30 boys) and 45 controls (23 girls and 22 boys) who completed scans. The mean ages of CWS and controls at the first visit were 5.55 (*SD* = 2.02) and 5.99 (*SD* = 2.00) years, ranging from 3 to 10 years. Another CWS was excluded due to incidental findings in the structural scan. Three CWS and one control were excluded due to excessive head movement during scanning.

### Final Data Set

The subject selection procedure described above is summarized in a flowchart (see Figure S1 in the appendix in the online supporting information located at https://www.mitpressjournals.org/doi/suppl/10.1162/nol_a_00017), and the demographics and behavioral results of the participants included in the final data set are listed in Table 1. The final analysis included 87 scans from 26 pCWS (8 girls and 18 boys; mean age at the first visit = 6.5 years; *SD* = 1.9), 61 scans from 17 rCWS (8 girls and 9 boys; mean age at the first visit = 5.4 years; *SD* = 1.9), and 139 scans from 44 controls (23 girls and 21 boys; mean age at the first visit = 6.5 years; *SD* = 2.0). These children were recruited from printed advertisements in newspapers or magazines (5 controls, 10 persistent, 7 recovered), parent email listservs (14 controls, 2 persistent, 1 recovered), study flyers (8 controls, 3 persistent, 1 recovered), SLP referral (1 control, 6 persistent, 1 recovered), word of mouth (5 controls, 2 persistent, 3 recovered), or other methods (e.g., Craigslist, Facebook, postcards; 2 controls, 3 persistent, 2 recovered). The specific ascertainment information was not known for 9 controls, 3 persistent, and 4 recovered, but all were recruited from the same general recruitment methods. Both persistent and recovered groups did not differ from controls in chronological age, sex, handedness, or socioeconomic status. Because *GNPTAG*, *NAGPA*, *GNPTG* and *AP4E1* are associated with persistent stuttering, only scans collected from pCWS and controls were included in the primary analysis of gene expression. Separate secondary analyses of pCWS (males only) compared to the control group, and rCWS compared to controls, were performed.

### Voxel-based Morphometry

Anatomical images were acquired on a GE 3T Signa scanner with an 8-channel head coil at Michigan State University. In each scan session, a whole brain three-dimensional inversion recovery fast spoiled gradient-recalled T1-weighted images with cerebrospinal fluid (CSF) suppressed was obtained using the following parameters: time of echo = 3.8 ms, time of repetition of acquisition = 8.6 ms, time of inversion = 831 ms, repetition time of inversion = 2,332 ms, flip angle = 8°, and receiver bandwidth = 620.8 kHz. For voxel-based morphometry (VBM) analysis, we used the optimized procedure proposed by Good et al. (2001). In summary, anatomical images were first segmented into different tissue partitions (Ashburner & Friston, 2005). Gray and white matter images were nonlinearly registered to a Montreal Neurological Institute (MNI) template using a diffeomorphic image registration algorithm (DARTEL) (Ashburner, 2007).

To accommodate for brain size differences, registrations were performed iteratively in a coarse-to-fine manner. Volumetric changes of each voxel were obtained by multiplying (or modulating) voxel values in the gray matter image by the deformation field derived from the registration procedure. Individual, modulated images were resampled to 1.5 mm isotropic voxels and spatially smoothed with a 6 mm full width half maximum (FWHM) kernel. To account for the dependence of participants’ multiple scans in this study, GMV images were analyzed using the sandwich estimator method, which was designed for analyzing longitudinal and repeated measures data (Guillaume, Hua, Thompson, Waldorp, & Nichols, 2014). The model included group (pCWS and controls) and group by age interaction as well as quadratic age, sex, IQ, brain size, socioeconomic status, and stuttering severity as covariates to control potential sources of variation. Although there was a significant difference between CWS and controls in IQ and both language measures, PPVT-4 and EVT-2 (Table 1), only IQ was included in the model because both measures were highly correlated with IQ (PPVT-IQ *r* = 0.70, EVT-IQ *r* = 0.69). The overall means of each covariate, except stuttering severity, were removed to capture the variation and potential differences between groups associated with the covariates. Since stuttering severity is only relevant to CWS, we considered stuttering severity to be zero for controls, and the mean for CWS was removed from the measure so that it would remove the variation associated with stuttering severity without affecting the group estimates.

Voxel-wise *t* statistics of the group difference were calculated. For comparing our VBM results with the findings in the literature, we also applied a threshold to visualize the significant GMV differences. However, identifying significant GMV differences between groups is not the primary goal of this study. A voxel-wise height threshold *p* < 0.005 and a cluster-size threshold *k* > 316 voxels were used to control for false positives. This set of thresholds corresponds to a corrected *p* < 0.05. The cluster-size threshold was determined by AFNI 3dClustSim (version 17.2.13; https://afni.nimh.nih.gov/pub/dist/doc/program_help/3dClustSim.html). Specifically, we first generated a non-Gaussian noise model according to the spatial smoothness of the residual images using the AFNI 3dFWHMx autocorrelation function (-acf option; https://afni.nimh.nih.gov/pub/dist/doc/program_help/3dFWHMx.html for 3dFWHMx). Then, we used Monte Carlo simulations implemented in AFNI 3dClustSim to estimate the false positive rate from the noise model (Cox, Chen, Glen, Reynolds, & Taylor, 2017).

### Gray Matter Volume and Gene Expression Correlation

Microarray-based gene expression data were obtained from the AIBS, which provides normalized expression of 29,131 genes using a total of 58,692 probes in each of 3,702 brain samples obtained from six adult donors (5 males, 1 female; age 24–57 years; see http://www.brain-map.org/ for details; Hawrylycz et al., 2012). We excluded genes whose symbols could not be identified in the HUGO Gene Nomenclature Committee database (https://www.genenames.org/), resulting in a total of 19,174 unique genes. The T1-weighted MRIs of the donors were segmented into different tissue partitions and normalized to the MNI template using the same procedure used for analyzing the structural images acquired from our pediatric subjects. Using the deformation field generated by DARTEL, the locations of brain samples in native space were transformed into the MNI space. The samples’ locations were mapped to 90 cortical and subcortical regions and the cerebellum, based on a standard atlas with automated anatomical labeling (Tzourio-Mazoyer et al., 2002). Because samples in the right hemisphere were taken from only two of the six donors, only supratentorial regions in the left hemisphere were included in our GMV-gene expression analysis. Since the right cerebellum has strong anatomical connections with the left cerebral hemisphere, and cerebellar anomalies have been associated with stuttering, the right cerebellum was included in our analyses as a single region. In total, 46 regions were included in the primary GMV-gene expression analysis. For each donor, expression of the same gene at each sample location from different probes was first averaged. Gene expression for each region was represented by the median of all the samples in the region. This step generated a parcellated expression map for each of the 19,174 genes.

The GMV difference of each region was calculated by taking the mean of voxel-wise absolute *t* statistics of between-group GMV differences (|*t* stat|) within the region. Absolute GMV difference was used because the effect of genetic variation on GMV in stuttering is not known. It is biologically plausible that the relationship between GMV and gene expression is directional. However, since the biological mechanism of this potential relationship is not known, we could not rule out the possibility that the relationship is nondirectional (i.e., the level of gene expression is only related to the magnitude of GMV difference). Thus, we chose to use the absolute GMV difference because it is sensitive to both possibilities (i.e., the directional and nondirectional relationships). However, this method ignores biologically plausible assumptions of directional correlations, that is to say, the higher the expression, the greater/lesser the GMV in CWS relative to controls. To further explore the potential directional relationship between gene expression and GMV differences, we carried out a post hoc analysis using the averaged *t* statistics across voxels in each region to represent the pattern of GMV difference.

As has been done previously in order to minimize the potential adverse effect of outliers, we used Spearman’s rank correlation to assess the relationship between gene expression and between-group GMV difference, instead of Pearson’s correlation (Ortiz-Terán et al., 2017; Richiardi et al., 2015). We calculated Spearman’s rank correlation coefficient (ρ) between GMV differences and each of the 19,174 genes expressed across the 46 regions. This procedure established a distribution of correlation coefficients. Statistical threshold was set at *q* < 0.05 (adjusted *p* < 0.05), corrected for multiple testing by controlling the false discovery rate (FDR; Benjamini & Hochberg, 1995).

### Gene Set Enrichment Analysis

Since genes other than *GNPTG* and *NAGPA* that are expressed in concordance with between-group GMV differences might also be associated with persistent stuttering, we carried out a gene set enrichment analysis to identify biological processes, molecular functions, cellular components, or KEGG pathways (Kyoto Encyclopedia of Genes and Genomes; https://www.genome.jp/kegg/) for which the top 2.5% of the genes that were most positively correlated with GMV differences are enriched. The 19,174 genes were used as the input of the background set for the enrichment analyses. We used PANTHER (http://geneontology.org/) to identify enrichment for biological processes, molecular functions, or cellular components, and DAVID (https://david.ncifcrf.gov/) for identifying enrichment for KEGG pathways (M. Ashburner et al., 2000; Huang, Sherman, & Lempicki, 2009; The Gene Ontology Consortium, 2017). The redundancy of the resulting gene ontology terms were removed by using REViGO (Supek, Bošnjak, Škunca, & Šmuc, 2011). Fisher’s exact test was used to determine statistical significance of enrichment factors. Statistical threshold was set at *q* < 0.05, corrected for multiple testing by controlling the FDR (Benjamini & Hochberg, 1995). Although the regional expression of our targeted four genes was only positively correlated with the GMV differences, for exploratory purposes, we carried out the same enrichment analysis using the 2.5% of the genes that were most negatively correlated with GMV differences.

## RESULTS

**Figure 1.**
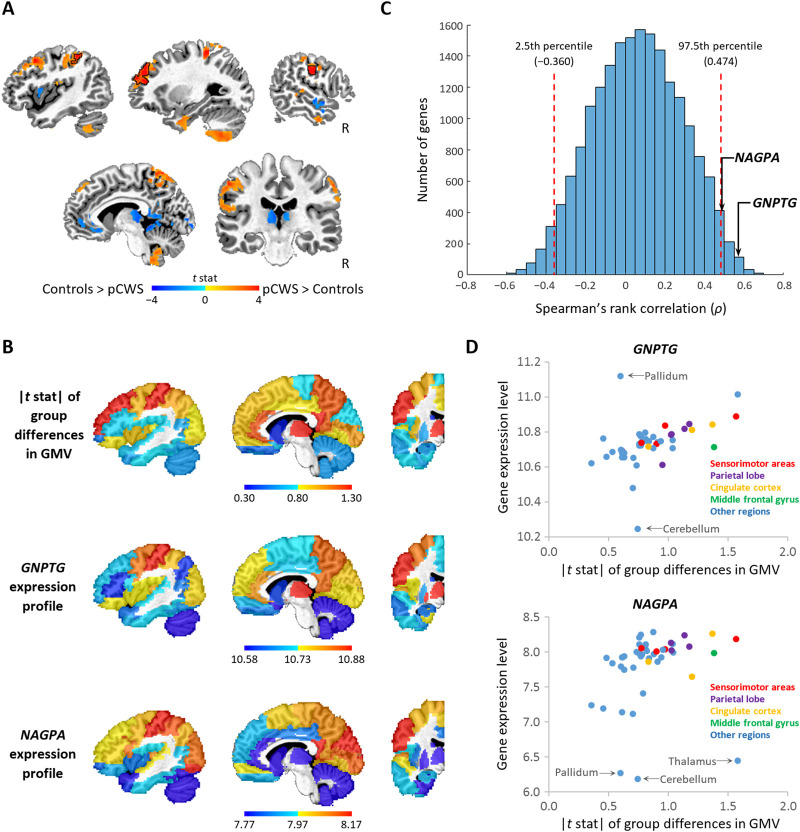
Spatial relationship between gene expression and between-groups differences in gray matter volume (GMV). (A) Voxel-wise differences between children with persistent stuttering (pCWS) and controls in GMV. Color-coded *t* values of group differences are overlaid on an anatomical image. Areas exhibiting a significant between-group difference in GMV (corrected *p* < 0.05) are outlined by black lines. The other colored areas are subthreshold (uncorrected *p* < 0.1). (B) Parcellated gene expression of *GNPTG* and *NAGPA* and absolute GMV differences in *t* statistics (|*t* stat|) in 45 left hemispheric regions and the right cerebellum (which anatomically connects to the left hemisphere) were overlaid on a single-subject anatomical image. The parcellation of the brain was based on a standard atlas with automated anatomical labeling (AAL). Gene expression and |*t* stat| of the right cerebellum were displayed in the left cerebellum to save space. (C) Frequency plot of Spearman’s correlation coefficients between GMV group differences and each of the 19,174 genes expressed across the regions. The red dashed lines indicate the levels of correlation at the 2.5th and 97.5th percentiles. The probability of obtaining a correlation >0.474 or <−0.360 is less than 5% if a gene is randomly selected. (D) Scatter plots between gene expression and group differences in GMV in the sensorimotor areas (red dots), the parietal lobe (purple dots), the cingulate cortex (orange dots), the middle frontal gyrus (green dots) and the rest of the regions (blue dots). Regions in which gene expression is 2.5 standard deviations above or below the mean are labeled.

We used 87 longitudinally acquired structural scans from 26 pCWS and 139 scans from 44 controls using VBM, a well-established neuroimaging technique (Ashburner, 2007; Good et al., 2001), to estimate voxel-wise GMV differences across the whole brain. Controlling for sex, age, quadratic age, cranial brain volume, IQ, socioeconomic status, and stuttering severity (SSI-4 score), the longitudinal analysis model showed that in the pCWS group, GMV in the left somatosensory, the left anterior prefrontal, and the right motor areas was significantly larger than in the control group (Figure 1A). Some regions such as the thalamus and the left inferior frontal gyrus showed a decrease in GMV, but none of them survived after correction for multiple comparisons (Figure 1A). We examined the spatial correspondence between the expression of the *GNPTG, NAGPA, GNPTAB* and *AP4E1* genes and between-group differences in GMV across the 46 regions using the Spearman rank correlation (Figure 1B). The correlation coefficients (ρ) associated with *GNPTG*, *NAGPA*, *GNPTAB* and *AP4E1* genes were 0.57 (*q* < 0.01), 0.49 (*q* < 0.05), −0.08 (*q* = 0.78), and 0.08 (*q* = 0.78), respectively. As illustrated in the frequency distribution for ρ for all 19,174 genes in our analysis (Figure 1C), the ρ values associated with *GNPTG* and *NAGPA* genes were significantly higher than the 97.5 percentile.

Since there was a tendency for a between-group difference in sex ratio, χ^2^(1, *N* = 70) = 3.09, *p* = 0.079, we conducted a follow-up analysis including only male pCWS and controls to rule out the possibility that the observed gene-brain relationship was driven by potential sex differences. The results of the male-only analysis were very similar to the original results (i.e., *GNPTG*, ρ = 0.47, *q* < 0.05; *NAGPA*, ρ = 0.57, *q* < 0.01; *GNPTAB*, ρ = 0.03, *q* = 0.93, and *AP4E1*, ρ = 0.01, *q* = 0.98), indicating that the results were not driven by a higher proportion of female subjects in the control group.

The scatter plots in Figure 1D show the relationship between gene expression and between-group differences in GMV across regions in the original analysis. We observed that the expression level of the *GNPTG* and *NAGPA* genes in the cerebellum and some subcortical regions, such as the pallidum, deviated from the relationship seen in the other regions. Repeating the analysis excluding the cerebellum, basal ganglia regions, and thalamus, we obtained similar results (i.e., *GNPTG*, ρ = 0.57, *q* < 0.05; *NAGPA*, ρ = 0.42, *q* < 0.10; *GNPTAB*, ρ = 0.02, *q* = 0.58, and *AP4E1*, ρ = 0.08, *q* = 0.50), although the correlation for *NAGPA* was no longer significant (see Figure S2 in the appendix in the online supporting information). Figure 1D further illustrates the monotonic relationship between the expression of GNPTG/NAGPA and between-group GMV differences in our original analysis. To further explore whether the relationship between gene expression and between-group differences in GMV was specific to persistent stuttering, we performed the same analysis on 17 rCWS. For rCWS, the relationship was not significant for any of the four genes (i.e., *GNPTG*, ρ = −0.08, *q* = 0.56; *NAGPA*, ρ = 0.20, *q* = 0.38: *GNPTAB*, ρ = −0.24, *q* = 0.34; *AP4E1*, ρ = 0.15, *q* = 0.45). Since we used the magnitude of between-group GMV differences to examine the spatial relationship with gene expression, the results reported above could not delineate whether the observed spatial correction was directional, for example, the higher the expression, the greater/lesser the GMV in CWS relative to controls. To explore whether the observed spatial correlation was directional or driven by the magnitude of between-group GMV differences, we carried out a post hoc directional analysis, which repeated the original analysis of spatial relationship using the directional GMV differences. We found no significant spatial correlation between the directional GMV differences and gene expression of the four genes associated with stuttering (*p* > 0.1).

Gene set enrichment analysis of the top 2.5% of the genes whose expression was most positively correlated with the between-group differences in GMV (see Table S1 in the appendix in the online supporting information) showed that this set of genes was highly enriched with genes involved in energy metabolism and mitochondrial functions (see Tables S2 and S3 in the online appendix). Genes in several KEGG pathways were also overrepresented in the gene set. Forty-nine out of 479 (10.2%) of the genes analyzed were involved in metabolic pathways, including overrepresentation of a subset of genes involved in citrate cycle (hsa00020) and oxidative phosphorylation (hsa00190; Table S4 in the online appendix). Genes involved in oxidative phosphorylation were also linked to a number of neurological disorders, including Parkinson’s disease, Alzheimer’s disease, Huntington’s disease, and amyotrophic lateral sclerosis (Table S5 in the online appendix). Using the same method, the top 2.5% of the genes negatively correlated with GMV differences were significantly enriched for the DNA packaging complex and the nucleosome (cellular components) as well as three KEGG pathways: alcoholism (hsa05034), systemic lupus erythematosus (hsa05322) and viral carcinogenesis (hsa05203; Tables S6 and S7 in the online appendix).

## DISCUSSION

To date, two VBM studies in children who stutter have been published in peer-reviewed journals (Beal, Gracco, Brettschneider, Kroll, & De Nil, 2013; Chang, Erickson, Ambrose, Hasegawa-Johnson, & Ludlow, 2008). Despite small sample sizes (<12 pCWS) and the use of relatively lenient statistical thresholds, both studies showed that smaller GMV in the bilateral inferior frontal gyrus (IFG) is associated with stuttering persistence in children. In the current study, decreased GMV in the IFG was observed only at an uncorrected threshold (Figure 1A). On the other hand, our data showed that stuttering persistence was associated with significantly increased GMV in the left prefrontal and bilateral sensorimotor areas, which have been shown to have increased blood flow during speech production in people who stutter in previous positron emission tomography studies (Braun et al., 1997; Fox et al., 1996). This finding is partially consistent with Beal et al. (2013), where pCWS exhibited increased GMV in right motor regions. From previous VBM studies in typically developing children, it is known that a majority of brain regions undergo GMV decreases during childhood, which reflects refinements of neural circuits via synaptic and dendritic pruning (Gennatas et al., 2017). The increased GMV in pCWS relative to controls in the present study may thus reflect a delay of development in those areas. Similarly, developmental delays in the structure of white matter tracts connecting speech-motor areas have been observed in a previous diffusion tensor imaging study using the same group of subjects (Chow & Chang, 2017). However, the connection between white and gray matter anomalies is unclear and warrants further analysis.

While our VBM analysis only showed significant GMV increases in pCWS relative to controls, some regions showed nonsignificant decreases in GMV. The magnitude of these differences (both increases and decreases) across regions in the left hemisphere and the right cerebellum was correlated with the gene expression patterns of *GNPTG* and *NAGPA*. However, when the directional GMV differences were used in the post hoc analyses, the spatial correlation with expression of all four targeted genes was no longer significant. The lack of directional correlation may limit the interpretation of our results. Taken together, our results indicated that the spatial relationship between *GNPTG*/*NAGPA* expression and GMV between-group differences was nondirectional, that is to say, primarily driven by the magnitude of GMV group differences. However, the biological mechanism(s) underlying the observed nondirectional relationship remains unknown. It is possible that gene expression is not the only factor associated with the pattern of GMV, but that the gene products may also have differential effects on GMV during various stages of neurodevelopment (Hoeft et al., 2010).

While the observed association between patterns of GMV differences and gene expression is correlational and does not provide direct evidence on the underlying casual mechanisms, here we discuss one potential biological mechanism that could explain the observed findings of the current study. *GNPTAB*, *GNPTG* and *NAGPA* are involved in the formation of the M6P tag that allows the binding of the lysosomal enzymes and other M6P-glycoproteins to the M6P receptor. While the main function of the M6P receptor is to transport the M6P-hydrolases from the trans Golgi network to the lysosomes, M6P receptors also bind other M6P-proteins and nonM6P proteins such as the insulin growth factor 2 (IGF2), a hormone that regulates cell metabolism and growth at the cell surface (Barnes et al., 2011; Gary-Bobo, Nirdé, Jeanjean, Morère, & Garcia, 2007; Han, D’Ercole, & Lund, 1987). The cation-independent M6P receptor plays an important role in the regulation of IGF2 levels by mediating its internalization and degradation (Oka, Kawasaki, & Yamashina, 1985). While IGF2 binds at a different site on the receptor than M6P tagged proteins, M6P lysosomal enzymes have been shown to alter the binding of IGF2 to the receptor (De Leon, Terry, Asmerom, & Nissley, 1996; Kiess et al., 1989). Thus, the altered binding of these enzymes due to genetic variations associated with stuttering might alter IGF2 mediated growth leading to altered GMV in regions where these enzymes are highly expressed. IGF2 has been linked to brain growth and differentiation as well as psychiatric and neurodegenerative disorders such as anxiety disorders and Parkinson’s disease (Fernandez & Torres-Alemán, 2012; Matrone et al., 2016; Pardo et al., 2018). While speculative, the connection between genes associated with stuttering and IGF2 would be an interesting future research direction.

The gene set enrichment analysis showed that genes expressed in concordance with the magnitude of between-group differences in GMV were significantly enriched for metabolic processes in mitochondria (see Tables S2 to S4 in the appendix in the online supporting information). Moreover, the gene set was also enriched for the KEGG pathways of a number of neurological disorders, including Parkinson’s disease, Alzheimer’s disease, Huntington’s disease, and amyotrophic lateral sclerosis (Table S4 in the online appendix). The genes involved in these disease pathways largely overlapped with genes involved in oxidative phosphorylation (Table S5 in the online appendix), suggesting that metabolic dysfunction similar to these diseases may play a role in stuttering. The positive spatial correlation between the magnitude of GMV differences and the expression of metabolic genes indicates that the regions exhibiting large GMV differences have higher energy metabolism rates (Goyal, Hawrylycz, Miller, Snyder, & Raichle, 2014). This result suggests that there may be a link between energy metabolism and the development of anomalous GMV in persistent stuttering. Metabolic genes could be directly related to persistent stuttering, but it is also possible that the GMV differences between pCWS and controls were exacerbated in brain regions with relatively high energy consumption related to disturbance in lysosomal function (McKenna, Schuck, & Ferreira, 2018). Future studies examining the participants’ genomes and their energy metabolism in the brain is necessary to further elucidate this relationship.

While our study showed a significant spatial relationship between the expression patterns of *GNPTG*/*NAGPA* and the magnitude of GMV regional differences between pCWS and controls, a few methodological caveats should be acknowledged. First, as mentioned above, a major limitation is that the genomes of our participants were not studied. Our results do not imply a direct causal relationship between genes and the brain anomalies, for example, that a mutation of *GNPTG* leads to GMV differences in people who stutter. To further understand our observed spatial relationships requires the examination of participants’ genomes and brain scans in the same individual. Second, although the sample size of the current study is one of the largest neuroimaging data sets in developmental stuttering and included repeated scans from each subject, it is small in the context of neuroanatomical studies examining correlates in complex heterogeneous traits. Small sample sizes could potentially lead to finding spurious group differences (Fusar-Poli et al., 2014), as well as lower power that limits detection of subtle differences that might otherwise be possible with larger samples.

Third, although gene expression data from AIBS were obtained from six adult donors, there is a high degree of similarity in the regional expression among donors (Hawrylycz et al., 2012). Moreover, the microarray gene expression data provided by AIBS were normalized within and across donors’ brains. The details of the current normalization method can be found in a technical paper from Allen Human Brain Atlas (http://help.brain-map.org/download/attachments/2818165/Normalization_WhitePaper.pdf). This normalization procedure reduces the variability across donors due to technical biases and allows comparison of expression data across two or more brains. However, a certain degree of variability between donors is inevitable. To further explore the individual donor variability, we examined the spatial relationship between the GMV differences and the expression of the four genes in each of the six donors. The correlation coefficients are presented in Table S8 (see the online appendix). In summary, we found that the correlation with *GNPTG* and *NAGPA* was positive for all six donors, and at least one was larger than 0.30 (*p* < 0.05) in four of the six donors, whereas the correlation with *AP4E1* and *GNPTAB* ranged from −0.23 to 0.20 (*p* > 0.1). The results of this individual donor analysis point in the same direction as our results using the gene expression aggregated from the six donors. For readers who are interested in further examining the individual donor variability, the regional expression patterns of the four targeted genes in each of the six donors are presented in Figures S3 and S4 (see the online appendix). To date, the expression data from AIBS are the only source of human gene expression patterns with high spatial resolution. As mentioned in the Introduction, AIBS gene expression data and the approach of the current study have been used to reveal relationships between genes and intrinsic brain networks, neuroplasticity-related genes, and altered functional connectivity in blind children, and the expression patterns of known risk genes and the brain anomalies of their associated neurological disorders including Alzheimer’s disease, Huntington’s disease, and schizophrenia (Grothe et al., 2018; McColgan et al., 2017; Ortiz-Terán et al., 2017; Richiardi et al., 2015; Romme et al., 2017).

Fourth, we cannot completely rule out the possibility that expression levels and patterns of some genes in children and adults are different. If this is the case for the genes associated with stuttering, we would expect that the spatial correlation between gene expression in the six adult donors and the GMV patterns in children would be weak, whereas a strong relationship was observed in our study. Moreover, previous studies have suggested that the changes in gene expression occur predominately during prenatal and infant development, and become relatively stable by around 6 years of age (Kang et al., 2011). Kang et al. estimated that only 9.1% of genes exhibit temporally differential expression in the first 20 years of life, and the portion of differentially expressed genes should be even less in our participants’ age group. Future studies using gene expression profiles in children should be pursued to refine our understanding of the relationship between brain anomalies and gene expression, when such data sets become available.

### Conclusions

In conclusion, we showed that relative to controls, pCWS exhibited greater GMV in the left somatosensory, the left anterior prefrontal, and the right motor areas. The nondirectional magnitude of GMV differences (both increases and decreases) in stuttering children relative to controls across regions in the left hemisphere and the right cerebellum was positively correlated with the expression of lysosomal targeting genes *GNPTG* and *NAGPA* as well as genes involved in energy metabolism. More research is warranted to further investigate possible roles of M6P mediated intracellular and extracellular trafficking, as well as whether metabolic functions play a role in the development of brain structural anomalies associated with stuttering.

## FUNDING INFORMATION

Ho Ming Chow, National Institute on Deafness and Other Communication Disorders (http://dx.doi.org/10.13039/100000055), Award ID: R21DC015853. Dennis Drayna, National Institute on Deafness and Other Communication Disorders (http://dx.doi.org/10.13039/100000055), Award ID: Z1A-000046. Soo-Eun Chang, National Institute on Deafness and Other Communication Disorders (http://dx.doi.org/10.13039/100000055), Award ID: R01DC011277. Soo-Eun Chang, National Institute on Deafness and Other Communication Disorders (http://dx.doi.org/10.13039/100000055), Award ID: R21DC015312. Soo-Eun Chang, Matthew K. Smith Stuttering Research Fund.

## AUTHOR CONTRIBUTIONS

Ho Ming Chow: Conceptualization, Data curation, Methodology, Formal analysis, Visualization, Writing—original draft, Writing—editing. Emily O. Garnett: Data curation, Writing—
